# Characteristics of Emergency Medicine Residency Programs in Colombia

**DOI:** 10.5811/westjem.2017.7.34668

**Published:** 2017-09-18

**Authors:** Andrés Patiño, Victor Alcalde, Camilo Gutierrez, Mauricio Garcia Romero, Atilio Moreno Carrillo, Luis E. Vargas, Carlos E. Vallejo, Virginia Zarama, José L. Mora Rodriguez, Yury Bustos, Juliana Granada, Leonar G. Aguiar, Salvador Menéndez, Jorge I. Cohen, Miguel A. Saavedra, Juan M. Rodriguez, Tatiana Roldan, Christian Arbelaez

**Affiliations:** * Harvard Affiliated Emergency Medicine Residency, Massachusetts General Hospital / Brigham and Women’s Hospital, Boston, Massachusetts; † Aria Health, Department of Emergency Medicine, Philadelphia, Pennsylvania; ‡ Boston Medical Center, Department of Emergency Medicine, Division of Pediatric Emergency Medicine, Boston, Massachusetts; § Asociación Colombiana de Especialistas en Urgencias y Emergencias, Barranquilla, Colombia; ¶ Pontificia Universidad Javeriana, Facultad de Medicina, Emergency Medicine Residency, Bogotá, Colombia; || Universidad del Rosario, Facultad de Medicina, Emergency Medicine Residency, Bogotá, Colombia; # Universidad de Antioquia, Facultad de Medicina, Emergency Medicine Residency, Medellin, Colombia; ** Universidad ICESI, Facultad de Medicina, Emergency Medicine Residency, Fundacion Valle del Lili, Cali, Colombia; †† Universidad de Caldas, Facultad de Medicina, Emergency Medicine Residency, Manizales, Colombia; ‡‡ Universidad CES, Facultad de Medicina, Emergency Medicine Residency, Medellin, Colombia; §§ Fundación Universitaria de Ciencias de la Salud, Facultad de Medicina, Emergency Medicine Residency, Bogotá, Colombia; ¶¶ Harvard Affiliated Emergency Medicine Residency, Massachusetts General Hospital / Brigham and Women’s Hospital, Boston, Massachusetts

## Abstract

**Introduction:**

Emergency medicine (EM) is in different stages of development around the world. Colombia has made significant strides in EM development in the last two decades and recognized it as a medical specialty in 2005. The country now has seven EM residency programs: three in the capital city of Bogotá, two in Medellin, one in Manizales, and one in Cali. The seven residency programs are in different stages of maturity, with the oldest founded 20 years ago and two founded in the last two years. The objective of this study was to characterize these seven residency programs.

**Methods:**

We conducted semi-structured interviews with faculty and residents from all the existing programs in 2013–2016. Topics included program characteristics and curricula.

**Results:**

Colombian EM residencies are three-year programs, with the exception of one four-year program. Programs accept 3–10 applicants yearly. Only one program has free tuition and the rest charge tuition. The number of EM faculty ranges from 2–15. EM rotation requirements range from 11–33% of total clinical time. One program does not have a pediatric rotation. The other programs require 1–2 months of pediatrics or pediatric EM. Critical care requirements range from 4–7 months. Other common rotations include anesthesia, general surgery, internal medicine, obstetrics, gynecology, orthopedics, ophthalmology, radiology, toxicology, psychiatry, neurology, cardiology, pulmonology, and trauma. All programs offer 4–6 hours of protected didactic time each week. Some programs require Advanced Cardiac Life Support, Pediatric Advanced Life Support and Advanced Trauma Life Support, with some programs providing these trainings in-house or subsidizing the cost. Most programs require one research project for graduation. Resident evaluations consist of written tests and oral exams several times per year. Point-of-care ultrasound training is provided in four of the seven programs.

**Conclusion:**

As emergency medicine continues to develop in Colombia, more residency programs are expected to emerge. Faculty development and sustainability of academic pursuits will be critically important. In the long term, the specialty will need to move toward certifying board exams and professional development through a national EM organization to promote standardization across programs.

## INTRODUCTION

Colombia is a country of 47 million people located in the northwest corner of South America. The largest cities are Bogotá (pop 8.7 million), Medellin (pop 3.5 million), and Cali (pop 2.4 million) (Figure).^1^ Despite a history of continuous internal armed conflict, Colombia has well-established democratic institutions and has made significant economic progress. In the last decade poverty has been reduced from 50% to 32.7%, extreme poverty has fallen from 17.7% to 10.4%, the capacity for basic education has been increased by almost 1.5 million, and unemployment has fallen from 15.6% to 9.6%.^2^

A new constitution in 1991 established healthcare as a fundamental right, and *Ley 100* [Law 100] of 1993 aimed to provide universal health insurance coverage. Although the level of health insurance coverage is high, access to healthcare varies greatly across geography, from small clinics with limited supplies and often staffed only by recent medical school graduates to tertiary hospitals in large cities, some with technology and resources like those of hospitals in developed nations.

In Colombia students apply to medical school immediately after high school.^3^ There are currently 58 medical schools, of which 69% are private and 31% are public.^4^ Medical school lasts 6–7 years, of which the last year, or *internado,* is similar in structure and responsibility to that of the first year of residency in the United States. After completing a year of service in an underserved area, graduates may apply to a residency, work independently as general practitioners in primary care, or work under the supervision of a specialist.^2^ Colombia has residencies in all specialties but spots are limited, admissions are very competitive, the positions are often unsalaried, and almost all charge tuition.

**Population Health Research Capsule**

What do we already know about this issue?

Few countries have described their residency programs in the literature, and there is no universal standard curriculum for emergency medicine residencies.

What was the research question?

What is the current state of emergency medicine residency programs in Colombia and what are their characteristics?

What was the major finding of the study?

The seven emergency medicine residency programs in Colombia have different training approaches.

How does this improve population health?

In comparing EM residency programs, this study promotes the standardization of residency curricula with the potential to impact emergency care in Colombia and other countries.

There is increasing demand for emergency medicine (EM)-trained providers in Colombia.^3^ Colombia has only about 200 trained EM specialists. In major urban areas most large hospitals have emergency physicians staffing higher acuity areas of the emergency department during part of the day. However, the great majority of emergency care is still provided by non residency-trained general practitioners and physicians from other specialties.^3^ The first EM residency in Colombia was founded in 1996. There are currently seven EM residency programs in the country (Table 1). The goal of this study was to characterize the current state of the seven EM residencies in Colombia.

## METHODS

Christian Arbelaez conducted site visits and semi-structured interviews with representatives from each of the seven EM residencies in Colombia between July 2013 and July 2016. Respondents included program directors, faculty, and residents. Phone calls and email communications were also used for follow-up questions. Topics covered in the interviews included the history of each program, number of residents, curricula, clinical sites, faculty, and challenges faced. Interviews were performed in Spanish, recorded, transcribed, and translated to English. We analyzed responses to create descriptions of each program and identify common themes. We also reviewed program websites and documents detailing curricula provided by the programs. This survey was granted exemption through the Partners Healthcare Institutional Review Board.

## RESULTS

### History of Emergency Medicine Residencies

Of the seven EM residency programs, three are in Bogotá, two in Medellin, one in Manizales, and one in Cali.^3^ The first EM residency program in Colombia was created in 1996 in Medellin by Universidad CES. In 2001 Universidad del Rosario in Bogotá, opened the second and only four-year program.^3^ In 2004 Universidad de Antioquia in Medellin started the only public, tuition-free program to date.^3^ In 2008, two programs opened in Bogotá: Pontificia Universidad Javeriana and Fundacion Universitaria de Ciencias de la Salud (FUCS). The Universidad de Caldas program started in Manizales in 2013. Universidad ICESI Fundación Valle del Lili in Cali was previously a site for the CES program and started its own residency program in 2016 (Table 1). In 2005, the Ministry of Social Protection (Ministry of Health) recognized EM as a medical specialty.^3^ There are currently two Colombian EM peer-reviewed journals. They include *Perspectiva en Urgencias*, from the Asociación Colombiana de Especialistas de Emergencias, and *Urgentia* from Javeriana. The Asociación Colombiana de Especialistas de Emergencias (ACEM) is the largest EM organization in the country, representing over 200 emergency physicians and EM residents.

### Applicant Selection

Applicants come mostly from the cities where the programs are located, but also from many other regions of the country. Similar to the application process for residencies in other specialties, EM residency applications are not centralized. The first step for all physicians applying to an EM residency consists of a general medicine written exam created by each residency program. Then each program has different processes to select candidates. CES conducts interviews with emphasis on clinical knowledge and leadership skills. Rosario has applicants shadow in the ED for half a day and discuss patient management with preceptors and also interview with psychiatry, EM faculty, the chief EM resident, the chief of EM, and an invited professor. Antioquia requires an English-language test and does not require interviews. Javeriana invites applicants with the best scores for a clinical simulation test, an oral exam and interviews with faculty and the program director. FUCS invites the applicants with the top five test scores for interviews with the program director, the assistant coordinator, a psychologist and human resources staff. Caldas and ICESI require interviews. Programs receive between 30 and 60 applications every year. Some programs accept new residents on a yearly basis: CES accepts six, Antioquia four, Caldas three, and ICESI four. The remaining programs accept new residents every six months: Rosario accepts five, Javeriana four, and FUCS three (Table 1). The graduation rate is 90–100% across programs.

### Tuition

Most residency programs in Colombia charge tuition. Antioquia is affiliated with a public university that does not charge tuition. Further, it provides a stipend of approximately $650 USD (CO$1,300,000) per semester. The rest of the programs do not provide a stipend and charge approximately $3,400 – $4,500 USD (CO$ 10,300,000 to 13,200,000) per semester (Table 1). *Crédito Ley 100* is a “forgivable” loan awarded to a limited number of residents through ICETEX, a government financial institution that provides financial aid for post-secondary education.^5^

### Residency Program Characteristics

The curricula of the different programs are loosely based on those of U.S. EM residencies but with significant variations (Table 2 and Table 3).^6^–^13^

### Emergency Medicine Faculty

At the time of this survey, the older programs, CES and Rosario, had the most EM faculty. CES had 15, and all EM rotations were done with EM faculty supervision. Rosario had 25 EM faculty, Javeriana nine EM faculty, FUCS two EM-trained faculty, and Antioquia had one full time and two part-time EM-trained faculty (Table 1). All program directors were EM-trained except for one who was surgery trained. EM is its own department at Rosario. At Javeriana and Antioquia EM is under the department of internal medicine.

### Point-of-Care Ultrasound Training

All Colombian programs cited point-of-care ultrasound (POCUS) as one of the weaknesses in their curricula in a 2014 study.^14^ The most commonly cited barriers to POCUS use were lack of instructors, lack of machines, and lack of time.^14^ Other barriers included turf battles with other specialties, billing issues and equipment cost. However, since 2014 POCUS has become more available and now Rosario, Javeriana, Caldas and ICESI offer ultrasound training.

### Residency Program Assessment and National Quality Assurance

The Ministry of Education plays an active role in ensuring the quality of postgraduate programs, including EM residencies, through the *Consejo Nacional de Acreditación de Colombia* (CNA), or National Accreditation Council. The accreditation system begins with a self-assessment, with the purpose of formulating actions to improve the quality of the program. This self-assessment is followed by an external evaluation by peer review, referred to as *Evaluaci*ó*n por Pares,* which evaluates the accuracy of the self-assessment and results in a submitted report to the CNA. Accreditation is granted after a final review based on the self-evaluation and peer review. This is valid for a period of 4–10 years depending on the quality of the program.^15^

### Post-Residency

Most EM-residency graduates are finding jobs in community hospitals or in academic centers, usually in critical care areas within the ED. Since the specialty of EM is relatively young, these graduates are often the first EM-trained physicians and are often in charge of establishing the specialty in those institutions. Fellowship training in critical care is available in some programs. Fellowships such as ultrasound, EMS, pediatric EM or disaster medicine are not currently available. Many EM residency graduates go on to work in intensive care units rather than EDs, given better financial incentives.

### Strengths and Challenges

#### CES

As the oldest program in the country with more than 20 years of experience, all EM rotations at CES are done with EM-trained faculty. The EM specialty and residency program are well established. Nonetheless, the program feels it needs to continue promoting itself within the university and hospitals to achieve the same level of recognition as older specialties.

#### Rosario

Rosario is the only four-year residency in the country and has 40 residents, the largest number in the country. Its seven clinical sites add expertise in trauma, toxicology, prehospital care, disaster preparedness, and cardiology. The program is working towards establishing a stronger academic connection with the university, since residents and faculty have felt disconnected from the larger university community.

#### Universidad de Antioquia

Antioquia is the only program in the country that offers free tuition. It has a strong emphasis on local epidemiology. Being part of the university faculty has significant financial benefits. Over the years the program has had to overcome political and administrative barriers within the hospitals and in relation to other specialties.

#### Javeriana

Javeriana has a strong emphasis on academic production and has its own academic journal. A weakness initially identified by trainees was the lack of ultrasound training. However, ultrasound training is now provided. Another weakness is relatively low exposure to trauma patients locally. However, at the time of this study, there was a plan to have residents do a trauma rotation at Hospital Universitario del Valle in Cali, which has large numbers of trauma.

#### FUCS

FUCS has a strong emphasis on critical care and the larger university has a strong tradition of academic training with one of its hospitals having had the first residencies in any specialty in the country more than 120 years ago. Two weakness identified by the program are its lack of ultrasound training and absence of a formal university affiliation for program faculty.

#### Caldas and ICESI

These two programs are new with a small faculty but have dynamic leaders as program directors. They are both located in urban settings and are affiliated with strong medical schools that offer excellent clinical training.

## DISCUSSION

Colombia is a land of contrasts. Its large cities have hospitals that rival those in the developed world, while healthcare in rural areas is more akin to that of a developing country with minimal infrastructure. EM professionals not only can improve the care Colombians receive in the ED but also bring expertise to strengthen prehospital and disaster care, both in urban and rural underserved areas. While Colombia’s Constitution of 1991 established healthcare as a right and Law 100 expanded health insurance coverage to cover greater than 90% of the population, access, quality and funding continue to be a challenge. Deficiencies in the system have led to ED crowding around the country. Most emergency care in Colombia is still being provided by non-residency trained providers. Emergency care requires expertise in the recognition and timely treatment of life-threatening conditions as well as the prioritization of resources for the flow of the ED. Now more than ever EM-trained physicians can help maximize ED resources to optimize throughput and clinical outcomes.

Colombia has made important strides in the development of EM with its seven residency programs and official recognition of EM as a specialty. Curricula are similar to those of residencies in the U.S., though with important variation. For example, ACGME requires 60% of clinical time to be spent in the ED under the supervision of EM-trained faculty.^13^ In contrast, EM residents in Colombia spend 11–39% of their time in the ED. This low ratio of EM clinical time is likely related to the youth of EM as specialty in Colombia and the relatively few EM faculty. Colombian EM residents receive strong training in critical care with programs requiring 4–7 months, compared to the 4-month ACGME requirement. All programs offer about 4–6 hours of protected didactic time each week and all programs require or encourage residents to obtain Avanced Cardiac Life Support (ACLS), Pediatric Advanced Life Support (PALS), and Advanced Trauma Life Support (ATLS) certifications. Ultrasound training has been expanding, with four of the seven residencies providing ultrasound training at this time. Ultrasound is Colombia is not only an important tool for every emergency physician, but it can also be crucial as EM-trained providers start working in hospitals in more rural areas with no other imaging resources.

Most EM residency graduates go on to work in community hospitals with most becoming the first EM specialist at their workplaces. Many go on to work in intensive care units given their extensive training in critical care and better compensation. As EM matures, the specialty must advocate for better compensation and working conditions in order to attract emergency physicians to EDs. As EM continues to develop in Colombia, more residency programs are expected to emerge along with a growing number of EM faculty. Standardization of training across programs, certifying board exams, strengthening of professional societies, and academic development will be important steps to further advance the specialty.

## LIMITATIONS

A limitation for this study is its data collection over a three-year span, which with the rapid evolution of the residency programs may have resulted in some of the results not being up to date at the time of publication. Co-authors of the study are part of the different residency programs, which could have introduced bias. However, this is balanced by the fact that each residency is represented by a co-author in the study.

## CONCLUSION

Colombia has made great strides in the development of EM. EM continues to gain traction as a specialty and the number of residencies will likely continue to grow. There are seven EM residencies at this time with different curricula that will serve as models for future programs.

## Figures and Tables

**Figure:**
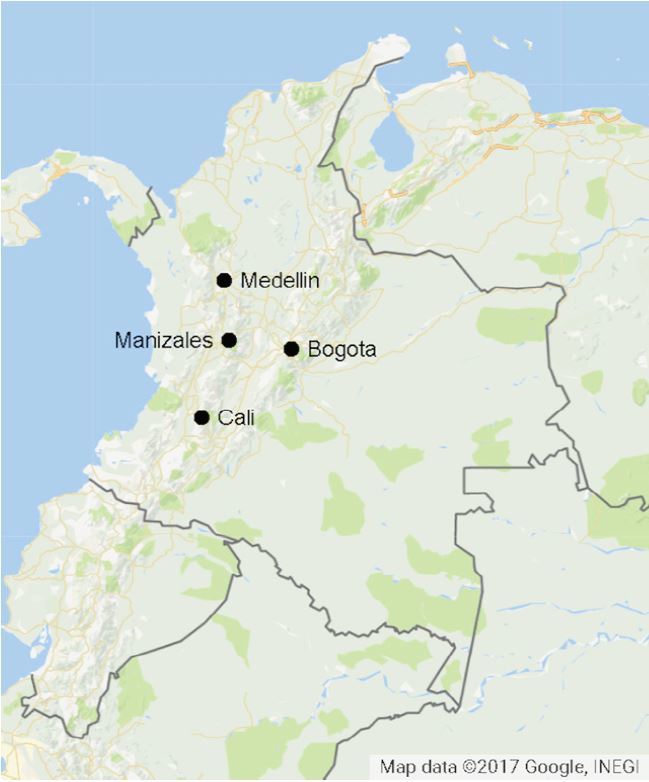
Map of Colombia. Emergency medicine residencies in Colombia are found in the three largest cities, Bogotá, Medellin and Cali as well as in the mid-size city of Manizales.

**Table 1:** General program characteristics of the seven emergency medicine residency programs in Colombia, which differ in tuition, length, number of faculty, number of residents and fellowships offered.

Program	CES	Rosario	Antioquia	Javeriana	FUCS	Caldas	ICESI
Year founded	1996	2001	2004	2008	2008	2013	2016
City	Medellin	Bogotá	Medellin	Bogotá	Bogotá	Manizales	Cali
Length	3 years	4 years	3 years	3 years	3 years	3 years	3 years
Tuition A	10,661,000	13,000,000	No tuition	13,228,000	13,050,000	10,300,000	11,840,000
Class size B	6	10	4	8	6	3	4
Residents total C	17	40	9	17	21	8	2
Emergency medicine faculty	15	10	1 plus 2 part-time	9	2	3	5
Application requirements D	Interviews	Interviews	English test	Simulation, oral exam, interviews	Interviews	Interviews	Interviews
Fellowships	Critical care	None	Critical care	None	None	None	Critical care
Special features	Oldest EM program in the country All EM rotations done with EM faculty	Only four-year program in the country	Only program with free tuition	Program has its own academic journal	Hospital San José was first hospital to train medical specialists in Colombia, starting 120 years ago	First residency program in a mid-size city	Newest program in the country

*FUCS*, Fundacion Universitaria de Ciencias de la Salud.

A Tuition in Colombian pesos (2,900 Colombian pesos ~ 1 USD).

B Number of residents accepted per year.

C Total number of residents in the program.

D Application requirements listed are in addition to a written test, which all programs require.

**Table 2:** Clinical rotation curricula In Colombia. Colombian emergency medicine (EM) residency curricula are loosely based on U.S. EM residency curricula with important differences in percent of time spent in EM and other rotations. Residencies in the U.S. are accredited by the Accreditation Council for Graduate Medical Education (ACGME). ACGME requirements for EM residencies are included in the table for comparison.

Program	CES	Rosario	Antioquia	Javeriana	FUCS	Caldas	ICESI	U.S. ACGME^13^
EM time in months	11 (31%)	11 (23%)	4 (11%)	7 (19%)	12 (33%)	10 (28%)	7(19%)	60% of all clinical time
Pediatric time in months	2	2	2	4	1	2	None	5 months (or 20% of all EM time)
Critical care time in months	4	7	4	2	6.5	6	6	4 (2 during PGY2 or higher)
Obstetrics time in months	1	2	2	2	1	1	1	0.5 months or 10 low risk vaginal deliveries
Other Rotations	Anesthesia Cardiology Surgery IM Neurology Orthopedics Psychiatry Radiology Toxicology Elective	Anesthesia Cardiology Surgery IM Neurology Orthopedics Pain Psychiatry Radiology Toxicology Trauma Ultrasound Elective	Anesthesia Cardiology ENT/ophtho Surgery ID IM Neurology Orientation Orthopedics Plastic Surgery Radiology Toxicology	Anesthesia Cardiology ENT/ophtho Surgery IM Orthopedics Ophthalmology Psychiatry Radiology Trauma Ultrasound Urology Health Administration Electives	Anesthesia Surgery Neurology Neurosurgery Orthopedics Pulmonology Radiology Toxicology ENT Ophthalmology Research Electives	Anesthesia Endocrinology Cardio/ Pulm ENT Gastroenterology ID IM Nephrology Neuro-trauma Ophthalmology Plastic surgery Psychiatry Radiology Surgery Toxicology Trauma Ultrasound Urology Electives	Anesthesia Cardiology Epidemiology ID Nephrology Neurology Neurosurgery Pulmonology Radiology Research Toxicology Trauma Ultrasound Elective	Varies by program. Anesthesia, orthopedics, toxicology, ophthalmology, ENT common. Ultrasound required

*ACGME*,Accreditation Council for Graduate Medical Education; *EM*, emergency medicine; *ENT*, otorhinolaryngology; *ID*, infectious diseases; *IM*, internal medicine; *Ophtho*, ophthalmology.

**Table 3:** Didactics, research, and resident evaluation. The seven EM residency programs in Colombia differ in didactics, research and resident evaluation requirements. The U.S. ACGME* requirements are included for comparison.

	CES	Rosario	Antioquia	Javeriana	FUCS	Caldas	ICESI	U.S. ACGME
Didactics (hours per week)	4 plus rotation-specific didactics	5 plus 4 hours of research	5	5	6	Varies	6	5
Prehospital rotations	Educational sessions	None	4-week rotation	1-month rotation	No information available	Educational sessions	None	Ambulance rides Direct medical command experience Multi-casualty drills
Certifications (ACLS, ATLS, PALS)	Done in-house	Program covers 60% of cost	Not required but encouraged	ACLS and ATLS required	Required	ACLS required	Not required	Not required by ACGME but required by most hospitals
Research Requirement	1 project	1 project /semester 1 final thesis	1 project	1 project	1 project / year	1 project	None	1 project
Evaluation	Written test at random times Individual evaluation for each rotation according to competencies	Written tests every 3 months Evaluations after each rotation Evaluation for promotion to following year (meeting with PD)	Evaluation at the end of each rotation Semester evaluation “German seminar” i.e. seeing patients with faculty and getting feedback	Written exams every 3 months by subject Written and oral exam at the end of each rotation Test for promotion to the following year	Written and oral exams every 3 months administered by internists and surgeons	Clinical supervisors evaluate residents after every rotation block on their knowledge, clinical skills, teaching skills, and bedside manner.	Evaluation at the end of each rotation	Continuous clinical evaluation Twice-yearly written feedback on clinical perforance Yearly evaluation with program director

*ACLS*, Advanced Cardiac Life Support; *ATLS* Advanced Trauma Life Support; *PALS*, Pediatric Advanced Life Support, *ACGME*, Accreditation Council for Graduate Medical Education; *PD*, program Director.
